# Assessing social contracts for urban adaptation through social listening on Twitter

**DOI:** 10.1038/s42949-023-00108-x

**Published:** 2023-06-05

**Authors:** Deepal Doshi, Matthias Garschagen

**Affiliations:** Ludwig-Maximilians-University Munich, Munich, Germany

**Keywords:** Governance, Climate change

## Abstract

Adapting to climate change impacts requires a coherent social contract in which different actors agree on a clear distribution of roles and responsibilities. An urgent requirement is to understand the imagined social contracts on expected roles and responsibilities, which is particularly relevant in cities where very diverse social groups come together. However, there is limited empirical evidence on these expectations as they are often tacit and hard to capture across large populations and heterogeneous groups. Here we assess the social contract on flood risk management in Mumbai, using the concept of social listening in combination with Twitter data. We find wide gaps between and within imagined social contracts. Sentiments such as frustration and apathy expressed in tweets explain these gaps and highlight the need to build trust for achieving accepted and effective social contracts for adaptation. Theoretical, empirical, and methodological lessons can be transferred to other cities and beyond.

## Introduction

The impacts expected from climate change will be so pervasive, that they will require stepping up adaptation efforts significantly, in many respects requiring fundamental transformations in the way societies manage their risks^1–7^. Cities, in particular, are faced with high adaptation challenges given that they are often at the frontline of climate hazard exposure^8^ whilst being characterized by high path-dependency, making transformative adaptation difficult and socially contested^3^. Meeting the stark adaptation challenges therefore will require collective efforts from different actors of society (state, citizens, civil society, private sector etc.), ideally with a shared understanding on common adaptation goals and clear distribution of tasks and responsibilities^9–13^. In reality, however, multi-actor constellations are often characterized by conflicting viewpoints on what actors expect from other actors or roles and responsibilities that actor groups ascribe to other actor groups. Related rifts and ambiguities have been identified as significant barrier in adaptation governance^14^. It is therefore important, first, to lay open and make explicit the often tacit or implicit viewpoints different actors have on their own as well as others’ roles and responsibilities regarding climate change adaptation, second, to assess how actors in cities and other social contexts negotiate potentially diverging viewpoints and, third, to examine whether and how they settle at an arrangement which helps to moderate unresolvable gaps in expectations and ideally arrive at a shared vision on how responsibilities for adaptation should be distributed^15–21^. However, this understanding is largely lacking to date, especially in urban settings where diverse social groups and their worldviews clash.

Previous literature has made important contributions to assess how adaptation goals as well as roles and responsibilities for adaptation are being negotiated—which forms the core of adaptation governance^22^. The notion of ‘social contracts’ has in this context been suggested in the literature, arguing that such a lens can guide future research to explicate the complex politics of adaptation^23^. Yet, only a limited number of studies on adaptation and related fields of sustainability, resilience or disaster risk management have used the term social contract^9,13,17,21,24–26^, and if so mostly in a loose and rather inexplicit or little conceptualized way. Most literature engages with topics around roles and responsibility for adaptation without explicitly referring to the notion of social contracts^10,14–16,18^. Also the latest IPCC assessment report, which is based on the available literature, does not explicitly assess social contracts for adaptation^22^. This shows that the concept has so far gained little traction, despite the presumed gains that it would hold for knowledge generation and decision support. Our study responds to the call for using social contracts as a stronger analytical lens^23^ and develop an approach to empirically assess social contracts for adaptation.

**Fig. 1:**
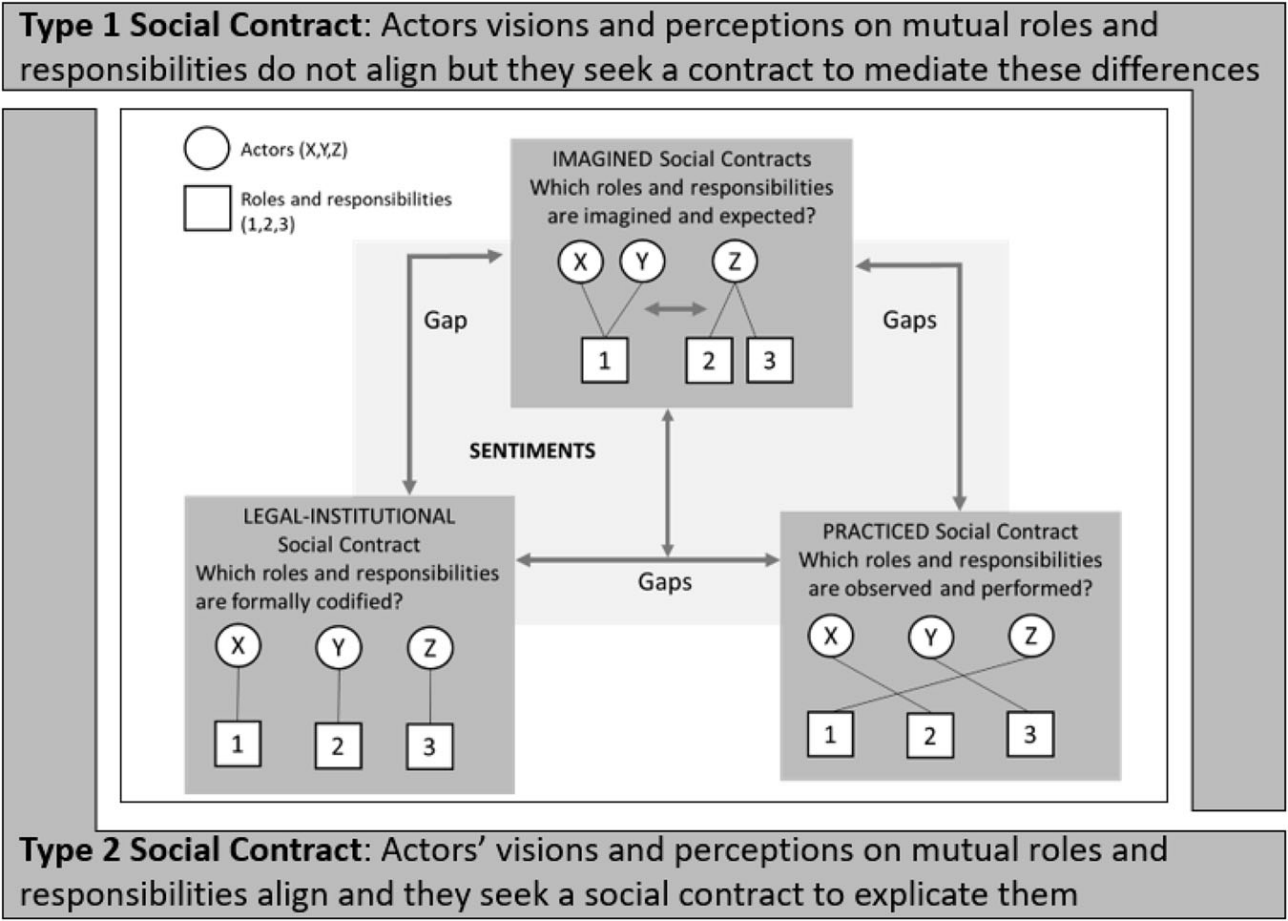
Conceptual framework showing the types, dimensions, and gaps in social contracts.

Building on literature on adaptation goals, risk governance and responsibility for adaptation^9–13^, we define a social contract for climate change adaptation as a collective arrangement between different actors of a society on the overall vision and goals as well as the mutual distribution of roles and responsibilities to achieve those goals. In other words, a social contract describes the collective arrangement of what a society wants and how it gets there. We conceptualize social contracts to be of two types (Fig. 1). Type 1 describes a social contract which exists where actors’ visions and perceptions on mutual roles and responsibilities do not align but where actors seek a social contract to precisely mediate these differences. Type 2 describes a social contract in a situation in which actors’ visions and perceptions on mutual roles and responsibilities align and actors seek a social contract to explicate and formalize this agreement.

Within each of these types (1 and 2), social contracts for adaptation can have three dimensions—imagined (ISC), practiced (PSC) and legal-institutional (LSC) (see Supplementary Table 1 for a detailed description)^23^. The ISC describes actors’ envisioned goals and viewpoints on the distribution of roles and responsibilities. The PSC describes the ‘real-life’ goals and observable (de facto) distribution of roles and responsibilities for adaptation between actors. The LSC describes the formally defined goals and visions and legally encoded (de jure) distribution of roles and responsibilities for adaptation between actors.

Our center stage for the empirical analysis of this paper is on understanding the imagined social contracts (see Supplementary Table 2 for detailed overview) and their relations to the practiced and legal social contracts. The imagined social contracts do not only result from the practiced and legal dimensions but also influence them. Hence, on the way towards encoding and practicing new social contracts, the most immediate need is a better understanding of the potentially diverging ways in which different actors envision new roles and responsibilities for other actors and themselves, i.e. which ISCs they have and wish for.

There may be gaps and contestations between the three dimensions of social contracts—for eg. rifts between the de facto, observable distribution of roles and responsibilities (practiced) and the de jure stipulations on formally defined roles and responsibilities (legal). Gaps could also exist within one dimension, e.g., when different actors have different imagined social contracts in mind regarding the distribution of roles and responsibilities. While it might not be possible to fully resolve these gaps and contestations, we suggest that engaging with these differences to at least identify them and become aware of them would allow actors to form a type 1 social contract to mediate the differences and deal with the gaps (which might still remain). Laying open these gaps and finding a way to deal with them would then ideally inform the process of actors aligning the gaps and potentially closing them with the objective of shaping a type 2 social contract.

Against this background, this paper aims to contribute to empirical knowledge on actors’ perceived roles and responsibilities, the potential gaps and contestations between them and the ways in which they are currently being negotiated. By doing so, the paper aims to inform the discussion and formation of at least type 1 and ideally type 2 social contracts on climate change adaptation in cities and beyond.

The need for explicit social contracts for adaptation is most starkly illustrated in cities, proving a valuable and apt unit of analysis. Different viewpoints on adaptation goals and priorities often clash in cities, as it is there that very heterogeneous social groups – characterized by socio-cultural diversity, competing economic and political priorities, asymmetric power relations, different levels of risk tolerance and adaptive capacities—are coming together^27^. These gaps and contestations may arise in view of addressing pertinent questions on political feasibility, power dynamics and trade-offs involved, such as whose priorities get embedded in adaptation pathways, who decides whose futures are protected and how costs are distributed, which spatio-temporal trade-offs will need to be made etc^28^.

We use the case study of flood risk management in the coastal megacity of Mumbai to assess the negotiation of social contracts for adaptation. Mumbai is the seventh largest metropolitan city globally and ranks among the top 10 coastal megacities at risk to coastal flooding and climate change impacts^29,30^ and hence is characterized by some of the highest adaptation pressure one can find^31–35^. While the city witnessed its most catastrophic flood event in 2005, when one-third of its annual rainfall fell in 24 hours resulting in the death of 1493 people and estimated losses of USD 1.7 billion^36,37^, heavy rainfall and flooding are almost an annual phenomenon during the monsoon season.

The current social contract for flood risk management in Mumbai is contested and riven between the practiced and legal social contract. Mumbai is confronted with stark inequality—being home to a powerful urban elite while 42% of the city’s population lives in informal settlements. The latter are at high-risk to flooding. Informal settlers are often being forced to live in environmentally risk prone areas, are socially excluded from civic services and poorer^38^, yet are often seen as illegal encroachments^39^. According to core national legislation^40^, disaster management responsibilities are entrusted to the state. Previous studies point out two major shortcomings in the legislation: one, the silence of the Act on state responsibility towards those impacted by disasters^41^ and two, the de facto implication of the Act on ‘active and willing support and cooperation of the local community’ in disaster management^42^. While the local municipal authority is entrusted with emergency response function, the national guidelines on urban flood management (UFM) foresee the role of citizens as first responders, even before state machinery steps in^43^.

The UFM guidelines recognize that the role of civil society has shifted from being “mere relief organizations to focusing on rehabilitation, reconstruction and mitigation” (p.101). Civil society is further explicitly expected to play a role in reducing socio-economic vulnerability of the poor^43^. Previous studies have emphasized the de facto role of civil society organizations in coping with flooding in Mumbai^44,45^. However, against the context of India’s economic liberalization which led to increased social and economic marginalization in major Indian cities, including Mumbai, it is important to note the dominant discourse on ‘civil society’ by urban elites which supports exclusionary restructuring policies against the poor^39^. A recent example of elite capture is seen in the contestations around the highly controversial Coastal Road infrastructure project which is perceived to serve the elite and has prevailed despite protests against it due to its adverse impacts on the sensitive coastline, livelihoods of fishing communities and being labeled maladaptive^46^.

Hence, there is an urgent need to capture the imagined, diverging viewpoints of different actors against this contested background. Furthermore, despite the freedom of speech being a constitutional right in India^47^, declining press freedom has been a concern raised in national and international media citing corporatisation in ownership, political control, safety of journalists and absence of civil society demands as the main reasons^48,49^. Therefore, in this analysis we explore social listening on Twitter, as it forms an increasingly important marketplace to capture different opinions and voices. However, in the context of debates on flood risk in Mumbai, elite actors potentially play a significant role due to favorable factors such as digital access and literacy.

In the age of digitalization and big data, social media, in terms of its volume, scale and speed offers many opportunities for urban sustainability research as well as urban planning and decision-making^50^. A wide range of quantitative (descriptive statistics such as correlation, regression, cluster analysis etc.) and qualitative (content analysis, social network analysis, thematic analysis etc.) research methods can be applied to different types of big data including Twitter^51–54^. Examples of studies using geotagged Twitter data, or sentiment analyses in urban, sustainability and adaptation research is manifold^51,55–58^.

**Fig. 2:**
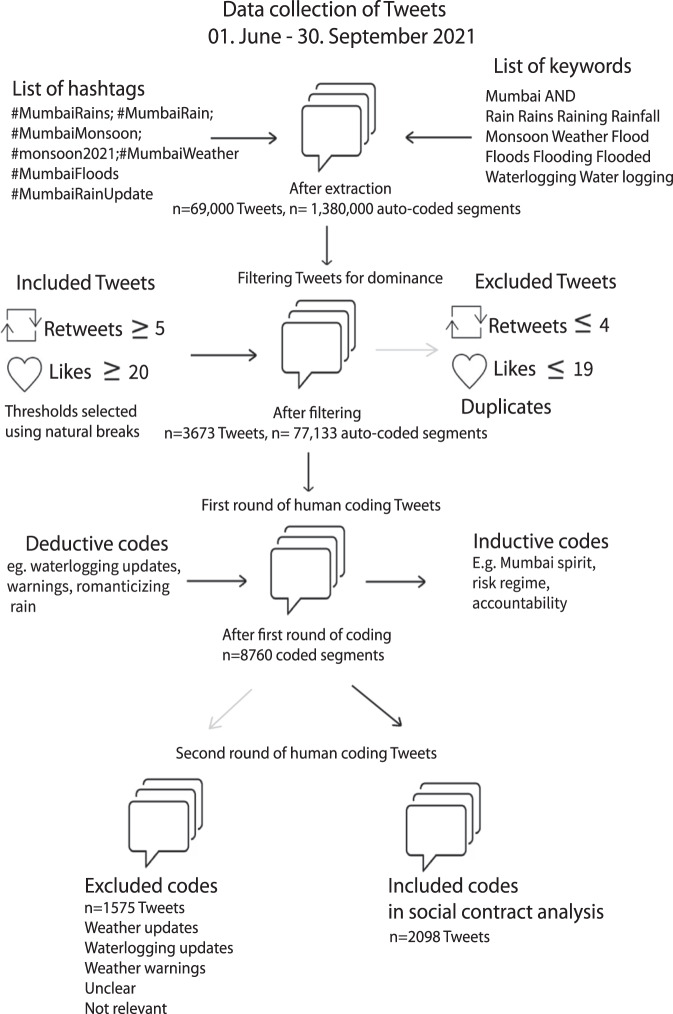
Flowchart of steps undertaken in Twitter data collection and analysis.

Social media offer an important arena to inductively capture and assess the exchange of opinions and negotiations of roles and responsibilities of different actors such as public sector, citizens, civil society and private sector, including nuanced sentiments such as frustrations, hopes etc. Adopting a grounded theory approach^59^, we combine the inductive exploration of data to capture the dominant debate on Twitter with a deductive application of a social contract theoretical lens. For this article, we developed and utilized the approach of social listening (also called social media analytics)^60^, defined as an “active process of attending to, observing, interpreting, and responding to a variety of stimuli through mediated, electronic, and social channels”^61^. Hence, the analysis strikes a balance in combining the potential of big data with context-specific insights to capture “contextual complexity” in adaptation research—as called for by Ford et al.^62^. Fig. 2 summarizes our workflow, a detailed methodology description is provided in the Methods section.

## Results

### Voices demanding better flood risk management and adaptation

**Fig. 3:**
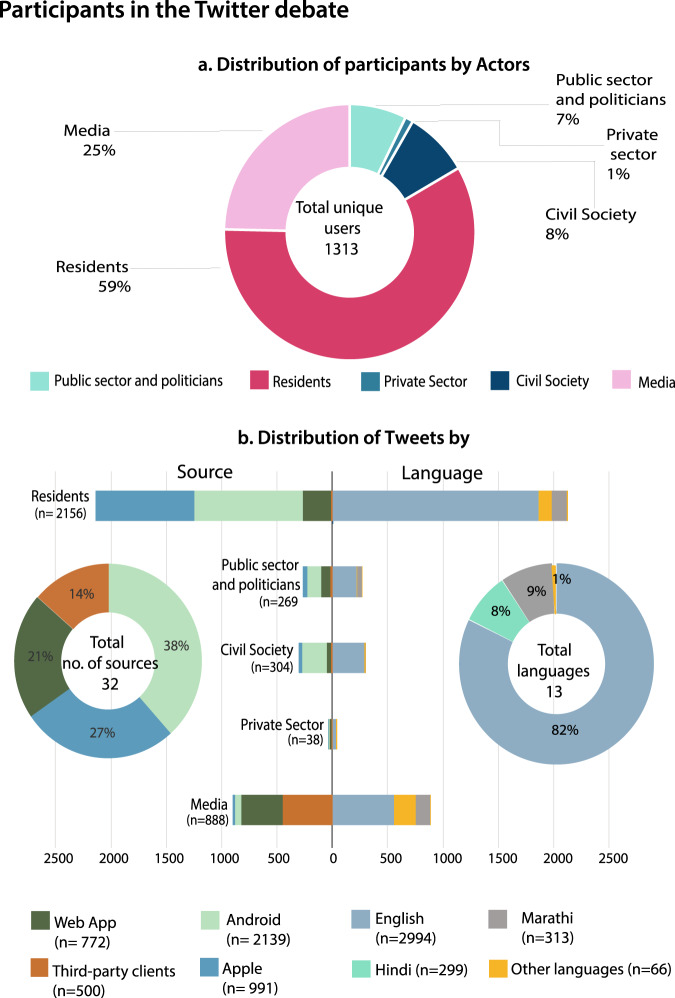
Profile of participants in the Twitter debate. **a** Distribution of Twitter users by actors; **b** Distribution of tweets by source device and language.

In terms of the overall Twitter user profile, our data shows that the vast majority of contributions to the flood-related debate in Mumbai comes from accounts held by private users / individuals (59%), most of which are likely to be residents of the city or otherwise closely connected to it, followed by accounts held by media organizations (25%), civil society groups, public sector organizations and politicians (e.g., civic authority, political parties, politicians such as ministers or mayors) and private sector (mainly private weather forecasting, insurance and aviation companies) (Fig. 3). The smaller number of tweets by actors such as civil society or public sector does not necessarily translate into a minor contribution to and influence over the debate, as many of these actors effectively serve as multipliers, e.g., in the case of political parties or civil society interest groups.

The participants of the dominant Twitter debate are largely composed of educated and affluent urban middle classes and elites, when measured along the Tweet’s language and type of device (Fig. 3). In India, the ability to communicate in English is strongly correlated with having a higher education level and economic status^63^. 82% of the tweets were posted in English, which compares to only 10% of the Indian population who speaks English. Even though the percentage of English-speakers is probably significantly higher in Mumbai—detailed numbers are lacking–this divide clearly indicates a dominance of the elite in the debate.

The picture that better-off actors participate over-proportionally in the flood-related Twitter debate also holds when looking at the type of devices used for tweeting (Fig. 3). 27% of the tweets in our dataset were posted from Apple devices, which compares to an India-wide market share of just above 3 percent for these devices^64^ even though the market share is probably higher in the city of Mumbai. Also, 38% of tweets were posted from usually more affordable Android devices, which compares to a national market share of almost 96% for these devices (ibid). Even though these figures suggest that better-off and affluent citizens contribute to the Twitter debate over-proportionally, it is important to highlight that many Twitter users raised their voice for others. Our data hence shows that especially marginalized and highly vulnerable groups who are not directly participating in the debate on Twitter are still strongly represented e.g., by civil society organizations.

**Fig. 4:**
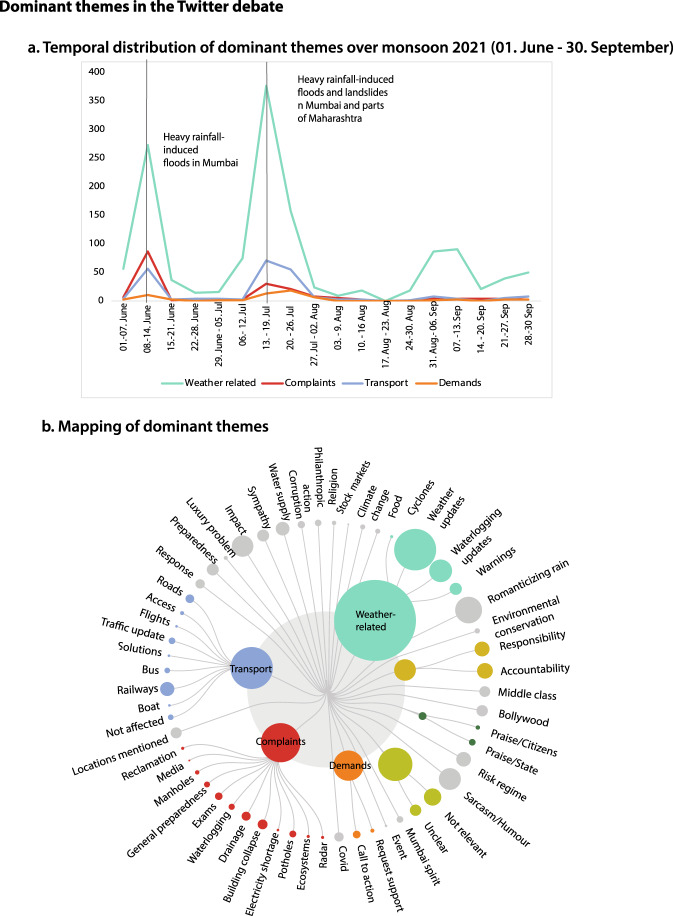
Dominant themes and trends in the Twitter debate on flood risk management in Mumbai. **a** Temporal distribution of dominant themes in the Twitter debate over monsoon 2021 (**b**) Mapping of dominant themes in the Twitter debate.

In terms of themes covered, our data shows that the Twitter debate covered a surprisingly wide range of topics, of which two-thirds of the tweets are in one or the other way relevant for the social contract analysis. Actors expressed their expectations of roles and responsibilities for flood risk management across a wide range of themes including transport-related concerns, complaints, impacts, demands etc. (Fig. 4b). One-third of the tweets mainly provided weather and waterlogging updates which provide relevant insights for flood hotspots mapping, early warning and emergency preparedness.

### Roles and responsibilities ascribed to the public sector

Expectations towards the public sector and politicians, in particular the ruling party and state bureaucracy, were mainly raised by individuals and the public sector itself i.e. opposition parties. Not surprisingly, our findings show those voices would aspirationally expect the public sector, especially the drainage department of the city’s civic authority, to be responsible for flood risk management. However, at the same time, our data clearly shows that in terms of realistic expectations—i.e. what the public sector will do rather than what it should do–actors did not anticipate the public sector and politicians to play that role in flood risk management. The analysis of sentiments proved to be an important lens for unpacking and understanding these gaps in actors’ expectations in more detail.

First, we find an important gap between the imagined and legal-institutional social contracts which help us understand why individuals and opposition parties ideally expect that flood risk management should be a public sector responsibility. We identified two major sentiments—frustration and humiliation—that explained this gap. Out of frustration, individuals and opposition parties claim accountability for the allocation of taxpayers’ money (Table 1, #1). Some voices even suggested to not pay taxes to the civic authority until the “waterlogging problem is solved” (Table 1, #2). This claim is also made in view of the allegations of corruption in drainage cleaning (legally a core task of the public sector^43,65^), as reported by mainstream media in the past years^66–68^. In addition, individuals expressed their humiliation when calling for public sector actors to take their responsibility for flood risk management more seriously. Tweets expressed feelings of shame, arguing that the city is facing flooding every year despite being the financial and commercial capital of the country and having the largest civic budget (Table 1, #3). However, surprisingly, none of the tweets called for specific adaptation or flood risk management measures. Instead, tweets tended to be more generic and called on the public sector and politicians to find solutions to “fix” the flooding problem (Table 1, #4).

**Table 1:** Example of Tweets supporting main findings

Roles and responsibilities		Tweet	Retweets	Likes
Roles and responsibilities ascribed to public sector and politicians	#1	Translated: In this city, one will find pits and holes, But even if you look, would not find the culprit #MumbaiRains #Monsoon2021 #Mumbai https://t.co/zlrunfCwmR	1256	10899
	#2	I personally feel, people of Mumbai shouldn’t pay taxes till every year’s rain water logging is sorted... Every year, same sight and no action to handle the situation for next year.. #Mumbai #MumbaiRainUpdate #MumbaiMonsoon	45	222
	#3	This won’t change. Now the incompetent politicians are blaming heavy Rainfall.what a shame. Is this the first tym this is happening? What happened to all the taxes collected by rich BMC? No solution all these years No solution for encroachments? #MumbaiRainUpdate #Chembur https://t.co/zJS3W6YslW	32	152
	#4	For decades, @mybmc has been unable to fix the flooding at King’s Circle, Matunga. See the situation today. And we are the richest civic body in the entire country. Vote these haftawallahs out in 2022. #MumbaiRains https://t.co/6SUs8zeIRa	61	290
	#5	Everything is covered in the name of Mumbai’s spirit during Mumbai Monsoons. It’s the survival and being left on your own. #MumbaiRainUpdate	28	250
	#6	“Lets hide every failure of BMC under the name of Mumbai Spirit https://t.co/N9x6BrmiIw #MumbaiRainUpdate”.	35	118
	#7	Saddening that over 25 people died due to wall collapses in Chembur &Vikhroli What is not acceptable is,if @OfficeofUT saw this coming,why was nothing done to evacuate people from low lying areas? #BMC,has been ruled by #ShivSena for 35+ yrs&yet this apathy? #MumbaiRainUpdate https://t.co/AEWXO4xhm9	121	411
	#8	Petition for basic survival support for homeless people coping with lockdown such as nutrition food & clean water. Dismissed by Mumbai High Court, saying homeless must work, not expect everything free. Extreme insensitivity: the homeless work very hard, else they wouldn’t survive https://t.co/8LJy62Qok	29	77
Roles and responsibilities ascribed to Residents	#9	“Mumbai’s middle class summed up: "As long as there are no potholes, no water and electricity cuts, Ola, Uber and Swiggys, and the trains run on time, why should we middle class people get involved in politics? Just work for some years and try and get US, UK or Aussie Citizenship"	36	128
	#10	“Mumbaikars will post about #MumbaiRains & related issues 30 times a year but won’t go out once in 5 years to vote out the corrupt nexus that has ruled Mumbai for 30 years. The Mumbai Spirit.”	433	1929
	#11	I think the so called Mumbai Spirit has made us insensitive. Immaterial what happens we have to keep working as if nothing happened. More than 20 ppl lost there lives in Mumbai due to rains & most are poor citizens. So obviously no outrage.”	45	152
	#12	To everyone who have problems with #MumbaiRains and pleading to stop every time it pours, fuck off and quit the city. I know cleanliness and flooding is a problem but the city is dependent on rains even for drinking water. So let it pour please.	10	61
Roles and responsibilities ascribed to Media	#13	Mumbai floods don’t make international news, but they still wreck lives. Like all climate disasters they will continue to get rapidly worse unless we stop adding greenhouse gases to the air. https://t.co/xCj7lk5GoK	108	223

Second, we clearly find a gap between the imagined and practiced social contracts which help us understand why individuals and opposition parties did not expect the public sector and politicians to play a role in flood risk management in reality. We identified two major sentiments—the lack of hope but also sympathy—that explained this gap. Individuals expressed their lack of hope in two ways: First, individuals and opposition parties showed their disappointment and frustration when the public sector and politicians do not deliver on their promises made before the monsoon. Individuals and opposition parties argue that apathy and ignorance on the part of the public sector and politicians in power explain this pattern. Second, many actors have given up hope because of experiencing flooding year after year. Individuals in particular felt being taken advantage of for their ‘everything goes’ attitude and the infamous ‘Mumbai spirit’ (which is used to praise the resilience of Mumbaikars)^69^ as an excuse by the public sector for their incompetence and poor governance (Table 1, #5, #6). Surprisingly, we identified a mix of humor and sarcasm to be a crucial sentiment in expressing and dealing with these gaps. Diverging from this view were a limited number of tweets, however, which also expressed sympathy for the public sector due to the intensity of rainfall.

Third, and probably most importantly, tweets revealed major contestations and gaps in the way different actors or even members of the same actor group (e.g., individuals) perceive and envision the imagined roles and responsibilities (ISCs) of public sector actors. These contestations most clearly surfaced in relation to the – often very laden – viewpoints on how to deal with the most vulnerable who often live in informal housing in high-risk areas. This debate peaked after thunderstorms and heavy rainfall led to landslides and collapsed walls, causing deaths of more than 30 people in mid-July 2021^70^, six weeks into the data collection (Fig. 4). In favor of the vulnerable, individuals, civil society, other political parties and politicians expressed sorrow and sympathy. Public sector actors responded to the incident by expressing condolences and announcing relief and compensation. Individuals and civil society expressed their anger and demanded (including a petition to the High Court) to relocate the most vulnerable to safer areas because they did not accept the status quo (Table 1, #7). Many individuals asked who was responsible for “this mess”, “these deaths”, “fallen houses, buried people, sunken cars” and “why was nothing done to evacuate people from low-lying areas”. In contrast, other individuals blamed the flood victims and demanded to remove them, arguing that they are illegal dwellers and taxpayers’ money should not be used for their rehabilitation. Supporting arguments to this narrative believed that such settlements were being protected by politicians due to their important role as vote banks, in line with other studies^38^. The High Court dismissed the petition for basic survival support for the homeless coping with lockdown, saying “homeless must work, not expect everything for free” as also reported in the media^71,72^ (Table 1, #8).

### Roles and responsibilities ascribed to individuals

Expectations towards individuals were primarily from individuals themselves and to a small extent from the public sector. Two diverging views could be identified. On the one hand, individuals questioned the responsibility of individuals and called out especially to the middle classes and the ‘dominant castes and classes’ to participate in politics and exercise their voting rights if they are not satisfied with the current leadership’s performance of flood management (Table 1, #9). Some individuals blamed other citizens of Mumbai for their ignorance and apathy under the disguise of ‘Mumbai spirit’ and humiliated fellow individuals for “watching this process for 25 years” for which they should be ashamed of. Similarly, some individuals questioned their impatience and lack of outrage given the high flood impacts and even casualties (Table 1, #10, #11). They perceived this as giving in to the alleged corruption by the public sector, arguing that corruption is one of the main culprits of ineffective drainage cleaning. Overall, many individuals blamed their fellow individuals to lack agency in the fight against the flood problem. Yet, others maintained that in times of “bad consequences” such as the “Mumbai rain”, it is the “people around you” that “come to help, not the politicians for whom you fight on social media everyday”, indicating many individuals already were active in self-help and others should become more active in that respect.

On the other hand, other individuals also asked fellow Mumbaikars to stop complaining about the floods in Mumbai. For example, media coverage of floods in the US and Europe at the same time as floods in Mumbai^73–75^ triggered some individuals to argue that Mumbaikars should not complain if even the developed world can be impacted by floods in such a drastic manner. Sympathizing with the public sector, some individuals believed that people should not be too critical of the public sector due to the intensity of rainfall. One view even asked all people who have a problem with “# MumbaiRains” to “quit the city” (Table 1, #12).

We identified only one major expectation from a politician to individuals, calling for the “rich and elite” to step forward and contribute to relief and support measures following the floods in Mumbai and parts of Maharashtra^76^. Other political parties expected individuals to be alert to “who really cares for them, who can solve their problems”.

### Role and responsibilities ascribed to the media

Individuals mainly expressed disappointment with the media and called for a stop to “doom and gloom” stories about Mumbai especially in view of the floods affecting the US and Europe as well. They also perceived international media as biased towards covering flood impacts of the US and Europe in comparison to “Mumbai floods” which “don’t make international news but still wreck lives” (Table 1, #13). This led individuals to believe that “some lives are more valuable” and question “why so many people in the US and Europe don’t care about the rest of the world”.

### Gaps hindering a new social contract for flood risk reduction

Overall, our results show that there are gaps in the social contract on flood risk management in Mumbai on two levels: first, between different social contracts such as the practiced and imagined or the legal-institutional and imagined and, second, between different imagined social contracts. On the first, we found a large gap between the aspirational (what actors should do or want them to do) and realistic (what they believed actors would actually do) levels of expectations towards the public sector. On the second, we found surprisingly stark contestations regarding the roles and responsibilities towards the poor and most vulnerable populations living in informal and highly flood-prone settlements. We also found similar gaps in expectations within the same actor group in other respects, most notably the levels of agency that individuals expect of fellow individuals.

Hence, our results show that there are not only wide gaps between the current de facto flood risk practices, the legal de jure stipulations and the envisioned flood risk management, but also between the social contracts imagined for the future, even within allegedly joint actor groups. These gaps are troubling in view of the already grave and intensifying flood problem, the heavy financial and human-resource costs of adaptation and the urgency resulting from long lead times of adaptation policies and actions. The results suggest that laying open these gaps is a necessary first step towards closing them and building a future social contract which helps mediate these differences and maybe/ideally form a coherent joint perspective.

## Discussion

We started our analysis by arguing that adapting cities to the inevitable impacts of climate change will require a strong and ideally coherent social contract in which different actors share an overall vision and agree on a clear distribution of roles and responsibilities for achieving that vision, despite potential differences in their respective individual perspectives. Our findings highlight the importance of improving our hitherto very patchy theoretical and empirical understanding of imagined social contracts in particular—and their relation to practiced and legal social contracts.

We found that there can be surprisingly large contestations and wide gaps regarding the roles and responsibilities which different actors envisioned for and ascribed to other actors. Our contribution is to lay open these gaps and disagreements in order to inform the discussion for actors to find an arrangement despite differences in viewpoints and arrive at a type 1 social contract. Ideally, we hope to even inform the process for actors to align the gaps and contestations—forming a type 2 social contract. Hence, through social listening, our findings show that becoming aware of these gaps between different expectations and decoding their drivers is the first step towards building new and coherent social contracts, so urgently needed for effective and equitable climate change adaptation across the globe^77^.

A social listening approach allowed us to capture unsolicited and therefore open views in a large-N sample and almost in real-time. Twitter is an increasingly important digital marketplace to negotiate and express opinions between different actors and hence, an important empirical database for analyzing evolving social contracts. The explicit analysis of sentiments expressed on Twitter turned out to be a useful tool which helped us to understand and explain the reasons driving disagreement on perceived roles and responsibilities between different actors. Combining big data approaches with manual qualitative coding allowed us to identify more nuanced sentiments such as apathy or frustration as compared to an algorithm trained classification of sentiments as positive, negative and neutral. This proved particularly helpful where expectations are tacit, i.e. where actors do not articulate them clearly and directly. Overall, the identified sentiments indicate a lack of trust between different actors and we suggest they might provide a helpful entry point into studying the formation of social contracts in other contexts or countries.

Despite the burgeoning potential of using social media data, a major limitation for this analysis is the representation of populations. Participation on social media is inherently linked to internet access and varies across geographies and demographics—also known as the “digital divide”^78^. Social media platforms including Twitter essentially allow to capture debates across trans-local networks, hence, the demographic composition of the actors in the Twitter debate in comparison to the demographic composition of the geographical population of Mumbai is not inherently problematic in our analysis. However, in the context of Twitter debates on flooding in Mumbai it may be important to bear in mind that urban elite are more likely to participate (in view of the dynamics discussed above) in comparison to informal, vulnerable populations. Nevertheless, civil society organizations, media, academics etc. still represent the concerns of the vulnerable populations.

In this study we do not aim to provide a complete assessment of social contracts in Mumbai through social listening, but capture an important segment of that debate taking place in the upcoming virtual space of Twitter. It is certainly not the only channel of information to understand the negotiation of social contracts and needs to be triangulated with other lines of information such as formal participation processes, informal discussions in local neighborhood groups etc. However, the importance of social media platforms is growing, especially with a growing middle class and India having the third largest Twitter community globally with its 23.6 million active users, preceded only by the USA and Japan^64^—making it a crucial case in Twitter research.

Social listening offers an important arena to capture dynamics of social contracts at unprecedented speed and scales, ranging from locally urban to global scales. Given the trans-local nature of social media debates, the approach used in this study could also be applied in other countries, albeit in view of the limitations and country context. For future research, we suggest, that using Twitter or other platforms of active exchange can be of great relevance in laying open gaps in high-risk contexts, including urban areas, in which different actors are faced with a high adaptation pressure and diverse competing, or even conflicting, perspectives but currently lack a clear and agreed strategy or even vision to jointly move adaptation forward^50,52,79^.

## Methods

### Data collection

In order to understand the imagined social contracts by different actors in Mumbai, we captured all flood risk related tweets over the monsoon season of 2021 (~70,000 tweets with 20 variables of metadata such as the author’s Twitter handle, number of re-tweets and likes, URL of the author etc. for each Tweet resulting in 1.3 million values of metadata). In a nutshell, we collected data through specific hashtag and keyword combinations and then filtered the results for dominance, with ~3600 dominant tweets defined through a high level of engagement in terms of re-tweets and likes. We then manually coded the tweets in order to show which actors participated and the major themes that emerged in the dominant debate on flood risk management. Subsequently, we filtered roughly two-thirds of the codes most relevant for the social contract analysis and coded and analyzed these tweets even more comprehensively (e.g., for underlying sentiments). In contrast to most studies which conduct quantitative sentiment analyses using Natural Language Processing (NLP) or other machine learning methods, we manually code sentiments to capture important nuances in the tweets (often multi-language tweets, context-specific words, memes and sentiments such as humor and sarcasm) to go beyond the positive, negative and neutral classifications generated by algorithms. We defined sentiments as feelings or emotions associated with viewpoints or opinions shared in a Tweet.

In a first step, we screened tweets on flooding in Mumbai in order to develop a list of the most popular hashtags and key-word combinations to capture the Twitter debate on flood risk in Mumbai. This list was revised in the first four weeks of data collection wherein hashtags which did not receive any hits were deleted and some new ones which gained popularity were added. The final list included: #MumbaiRains, #MumbaiRain, #MumbaiMonsoon, #monsoon2021 AND Mumbai, #MumbaiWeather, #MumbaiFloods, #MumbaiRainUpdate, one of the words (Rain Rains Raining Rainfall Monsoon Weather Flood Floods Flooding Flooded Waterlogging Waterlogging) AND Mumbai. Tweets were extracted using the Twitter API of MAXQDA. The results of the tweets extracted from MAXQDA’s Twitter API were compared to tweets extracted using Twitter’s Academic access API through R. Given no difference in the tweets, MAXQDA’s Twitter API was continued as it would allow easier synchronization for qualitative coding in future steps of the analysis.

Tweets were collected for a period of 4 months from 01 June to 30 September 2021—corresponding to the monsoon season in Mumbai. The monsoon season of 2021 was preceded by Cyclone Tauktae in May and India’s worst Covid-19 wave of the Delta variant from March to May. During the monsoon months of 2021 there were two major flood events in Mumbai and in other parts of Maharashtra. The floods in Mumbai also coincided with floods in Europe and in USA. The debates on Twitter are generally very open, in line with the long tradition of the country’s free speech and backed by The Indian Constitution which guarantees all citizens the fundamental right of “Freedom of speech and expression” in Article 19^47^.

A total of ca. 69,000 Tweets with around 1.3 million auto-coded segments were collected over these 4 months. Each Tweet text extracted using the MaxQDA API comes with 20 variables of metadata for example “Date and Time” when the Tweet was posted, “Author” name of the user on Twitter, “Author description” which is the self-description of the user on Twitter etc. Table 2 below shows the auto-codes during data extraction and how they were used in the analysis.

**Table 2:** Auto-coded metadata variables extracted for each Tweet using the Twitter API on MaxQDA.

	Auto-coded metadata by Twitter API in MaxQDA	Example	Use in the analysis
1	Date and Time	26.07.2021 15:03:36	Timeline analysis of tweets
2	Tweet	Maharashtra floods: Mumbai has some of richest people in world, they should help, says Sanjay Raut | Mumbai News—Times of India https://t.co/R8kMjbc0GE	Qualitative analysis for theme and sentiment
3	Hashtags	-	
4	Type	Tweet	Analysis focused on tweets, not re-tweets and replies
5	Answer to	-	
6	Author	rautsanjay61	Used to determine ‘Actor’ type in the dominant debate
7	Real name of author	Sanjay Raut	
8	Author place	Mumbai, India	
9	Author time zone	-	
10	Author URL	https://t.co/RHtudzxG0u	
11	Author description	Executive Editor, Dainik Saamana. Member of Parliament, Rajya Sabha. Shiv Sena Leader.	
12	Followers	835095	
13	Following	261	
14	Tweets	1891	
15	Profile verified	True	
16	Profile created	13.12.2013 15:28:05	
17	Retweets	290	Threshold of 5 and more for filtering dominant debate
18	Likes	2397	Threshold of 20 and more for dominant debate
19	Language	English	Proxy indicator for socio-economic status of user
20	Source	Twitter for Android	
21	Tweet coordinates	-	

### Data filtering and coding

Tweets were filtered for capturing the dominant debate. Dominance was operationalized in terms of level of engagement (re-tweets and likes). Based on natural breaks in the data, the threshold for dominance was set at 20 likes AND 5 re-tweets to qualify as a “highly dominant Tweet”. Hence, the dominant debate comprised of tweets that fulfilled both criteria since re-tweets and likes also showed a strong positive correlation. After filtering for dominance and removal of duplicates, the first database for manual coding comprised of 3673 tweets with 77,133 auto-coded segments.

The first round of coding generated 8760 coded segments with 29 primary-level codes and 33 secondary level codes. We found that two-thirds of the dominant debate (2098 tweets) comprised of primary-level codes such as transport, complaints, impacts, accountability etc. which are relevant for the social contract focus of the analysis in this paper. One-third of the debate (1575 tweets) were on weather related updates such as waterlogging, warnings etc. providing valuable information on flood hotspots and early warning communication. In a second round of coding tweets were further clustered according to roles and responsibilities ascribed to different actors: the public sector and politicians, individuals and media. A qualitative codebook is provided in Supplementary Table 3.

## Supplementary information

Supplementary Information

## Data Availability

The datasets generated during and/or analyzed during the current study are not publicly available but are available from the corresponding author on reasonable request.
