# Supplementary material for: Assessing social contracts for urban adaptation through social listening on Twitter
Source: NPJ Urban Sustain. 2023 Jun 5;3(1):30. doi: 10.1038/s42949-023-00108-x (PMC10243091; doi:10.1038/s42949-023-00108-x)
Supplement: Supplementary file 1 — Supplementary Information [file 42949_2023_108_MOESM1_ESM.pdf]

SUPPLEMENTARY INFORMATION

Supplementary table 1 Main characteristics relevant the analytical application of the three forms of social contracts – Imagined, Practiced and Legal-institutional social contract

	**Imagined Social Contracts (ISC)**	**Practiced Social Contract (PSC)**	**Legal-institutional Social Contract (LSC)**
**Rationale for why we need to talk about these**	Provides the opportunity to lay open the heterogeneous/diverse/diverging viewpoints which are often implicit, tacit and difficult to capture	Allows an understanding of the existing distribution of roles and responsibilities (de facto)	Helps to identify the legally defined roles and responsibilities held by different actors (de jure)
**Definition**	Describes actors' envisioned goals and viewpoints on the distribution of roles and responsibilities	Describes the "real-life" goals and observable distribution of roles and responsibilities between actors	Describes the formally defined goals and visions and legally encoded distribution of roles and responsibilities between actors
**Core question in this analysis**	Which roles and responsibilities are imagined and expected?	Which roles and responsibilities are observed and performed?	Which roles and responsibilities are formally codified?
**Forms of expression**	Perception ("this is what I believe it to be"/ what they think the actor will do); expectation ("this is how it should be"/ what the actor should do); hope/aspiration ("this is how I wish it to be"/what they hope or think the actor can be doing)	Observable in "real-life" and performed in everyday settings	Defined through formally codified in legal-institutional and constitutional frameworks
**Existing space**	Implicit/ tacit/imagined space	Material space	Formal codified space

Source: developed from Blackburn and Pelling 2018

Supplementary table 2 Selected aspects of Imagined Social Contracts (ISCs) and their relevance for adaptation

**Relevance to adaptation in general**	Helps to identify gaps and contestations in subjective viewpoints of adaptation goals and the negotiations of the roadmap to achieve them – especially those which do not get reflected in "practice" (PSC) or "policy" (LSC)<br><br>Opens the debate on socio-cultural limits to adaptation and boundaries of risk tolerance and acceptability<br><br>Analytical space to make explicit the discussion and negotiation of trade-offs – that there will be winners and losers in adaptation.
**Relevance to this study &**	ISCs help to capture diverging viewpoints in the debate and potential gaps and contestations between different actors or within the same actor group<br>ISCs constitute the core analytical space to capture the envisioned goals and roles and responsibilities for flood risk management
**Relation to social listening**	Qualitative analysis of sentiments allows to reveal that there might be gaps and contestations in the first place<br>ISCs are changing and social listening allows to capture those changes at a rapid speed because of the speed of data collection and possibility to do long term research.<br>Social listening provides an unsolicited approach to capturing 'unbiased' ISCs<br>"Digital divide" and ISCs – whose voices get heard – how does social media shape ISCs?
**Relation to PSC**	Gaps between ISCs and PSC shows whose priorities and values are embedded in the real world, in other words highlighting power relations<br>Whereas closeness to PSC may indicate capacity for citizen-led action to leverage priorities for adaptation
**Relation to LSC**	Closeness between ISCs and LSC may also help to trace where the calls for legally codified roles and responsibilities came from.<br>Gaps between LSC and ISCs may indicate complacent citizenship (for eg. due to political apathy).
**Relation to PSC/LSC**	It may or may not be reflected in PSC or LSC.<br>Provide space to analyse the negotiation of moral claims and obligations which may also play an important role in influencing LSC and PSC.<br>PSC is seen as "the product of negotiation between multiple conflicting ISCs (which may coexist) and the LSC, and may sit closer to one, both or neither"

Source: developed from Blackburn and Pelling 2018

Supplementary table 3 Qualitative codebook

Axial code	Primary code	Description	Example
Religion		This code applies to Tweets which invoke spiritual or religious intervention, offer prayers in support of individuals affected by floods or address a God in relation to monsoon.	A tribute to the monsoon woes of #Hindmata waterlogging at Parel.<br><br>Franklin Paul & friends recreated Hindmata area in the Ganpati Decoration.<br><br>Pic courtesy Ratik Chorge @HTMumbai #MumbaiWeather https://t.co/ASDw4Q37OJ
Stock markets		Tweets which link trends in the stock market to monsoon and flooding. Mumbai houses the headquarters of the National Stock Exchange of India.	Mera desh badal Raha hai<br>Aagey bad Raha hai.<br>#MumbaiRains #StockMarket<br>#nifty #sensex #traderslife #nseindia<br>https://t.co/56V7fVM9f3
Climate change		Tweets which allude to the impacts of climate change on Mumbai	#Mumbai municipal commissioner Iqbal Singh Chahal says by 2050, a major portion of south Mumbai, including the business district of Nariman Point and state secretariat Mantralaya, will go underwater due to #RisingSeaLevels ⤵<br>https://t.co/FeV6QzYjRp
Food		Tweets which refer to food that is especially related to monsoons and rains in Mumbai.	Thing's that can't be separated in Monsoon! #MumbaiRains<br><br>Bhajiya & Chai<br><br>BMC & Open Manhole https://t.co/LduYkKtXTe
Weather updates	Cyclones	Tweets that provide updates related to cyclones which may have an impact on Mumbai. (This dataset excludes the Tweets related to cyclone Tauktae which struck Mumbai in mid-May 2021, since Tweets were collected corresponding to the official monsoon period in Mumbai between June-September)	#CycloneGulab will make start making landfall by 8pm over #tekkali in Srikakulam district, AP. It will turn into a deep depression later. #mumbairains to increase from Mon 11 pm. If it will be mod to heavy OR heavy to very heavy will depend on how close it passes from #Mumbai https://t.co/1LoCLkLK1t
	Weather updates		
	Waterlogging updates		
	Warnings		
Romanticizing rain		This code is applied to Tweets in which actors romanticize the rain and monsoon in Mumbai. This code is often in relation to expressing the natural	Overcast but Beautiful<br>.<br>.<br>My Beautiful city with dark grey monsoon clouds in the background<br>.<br>The Bay , Mumbai

		beauty of the rain, links to Bollywood, enjoying certain food items in the monsoon rain etc.	.<br>#mumbai #mumbairains #artdeco #sea #view #sky #monsoon #weekend #weather #nft #NFTartist https://t.co/aUwGj5dbUA
Environmental conservation		Tweets which call for protection of biodiversity and ecosystems and impact of environmental destruction on flooding.	This is exactly what happens when we chop down trees, concretize every nook and corner.......& then paint trees & grass on a wall with a message......'Plant Trees and Save Trees'.<br>#Mumbai will keep flooding<br>#ClimateCrisis<br>#ClimateAction<br>Pic @diptivsingh https://t.co/5yWfRnoYCG
Social contract	Social contract	This code applies to Tweets which directly raise concerns or questions in relation to roles and responsibilities for flood risk management.	Tragic to hear of so many rain related deaths in Mumbai. Happens every year, deaths are reduced to a statistic. It seems max city is less prepared to face upto the brunt of the rain waters.. houses on hillocks live on edge year after year with no solution in sight. Prayers!😭
	Accountability	This sub-code applies to Tweets which raise questions of accountability for the perceived roles and responsibilities by actors.	Whole Mumbai is flooded with rains & incompetence corrupt BMC management instead of visiting war room & taking charge CM @OfficeofUT along with Rashmi Thackeray is enjoying long drive to Pandharpur.<br><br>Govinda Govinda!!
Middle class		This code refers to Tweets which describe the view of the middle classes in Mumbai.	Mumbai's middle class summed up: "As long as there are no potholes, no water and electricity cuts, Ola, Uber and Swiggys, and the trains run on time, why should we middle class people get involved in politics? Just work for some years and try and get US, UK or Aussie Citizenship"
Bollywood		This code applies to Tweets that make references to Bollywood actors or the industry in its role towards flooding, share pictures of Bollywood actors in relation to monsoon, share film references to the Mumbai monsoon.	How come after such heavy “rain drops” not a single celeb posting or tweeting abt the situation in mumbai?<br>Or all those post r script written just like their dialogues n all done only for money!<br>Where is the love for amchi mumbai?
Praise	Citizens	This sub- code refers to Tweets which praise civil society for their efforts towards dealing with the impacts of flooding.	Nothing can stopped us Team #SharingandCaring #fooddistribution #miraroad #mirabhayandar thank you all for your constant support 😭 #MumbaiRains #mumbairain #Mumbai #covidhelp https://t.co/35L3XU6vqk
	State	This sub-code refers to Tweets which praise state actors for their support and response in dealing with the impacts of flooding.	Taking a moment to thank @MumbaiPolice for their relentless work especially during #Mumbai rains ☔<br>Year after year, we see them at traffic lights, drenched, helping citizens, rushing victims to hospitals. Do you stop to even smile at them? #India 🇮🇳 https://t.co/oqaIUkUJW9

Risk regime		This code refers to Tweets which express opinions on risk management regimes, transitions or the lack thereof.	For decades, @mybmc has been unable to fix the flooding at King's Circle, Matunga. See the situation today.<br>And we are the richest civic body in the entire country.<br><br>Vote these haftawallahs out in 2022. #MumbaiRains https://t.co/6SUs8zeIRa
Sarcasm/ Humour		This code is applied to Tweets that particularly express the sentiment of using sarcasm and/or humour in stating their view on flood risk management.	Delhi & Mumbai flooding due to swindling of funds meant for drainage system.<br>Kejriwal & Thakre are trying to give a look of Venice to Delhi & Bombay 😎🤣
Excluded	Not relevant	This code refers to Tweets which may have made it into the dataset because of the keyword and/or hashtag filter but are not relevant to the debate on flood risk management in Mumbai	#StopEatingMeat #Bakrid #EidAlAdha #BakriEid #MumbaiRains #HeavyRains #NelsonMandelaDay<br><br>Hey #PETA (@peta & @PetaIndia), Bakrid is coming. Are you afraid to talk about animal cruelty on Muslim festivals ? #BakraLivesMatter https://t.co/cEkTHjj5hi
	Unclear	This sub-code refers to Tweets which may have made it into the dataset because of the keyword and/or hashtag filter but their exact relation to the debate on flood risk management in Mumbai is unclear to the authors.	जो मुँह तक उड़ रही थी, अब लिपटी है पाँव से,<br>बारिश क्या हुई मिट्टी की फ़ितरत बदल गई ....<br>#MumbaiRains #Monsoon2021<br>Translated by Google: The one who was flying till the face, is now wrapped around the feet,<br>It rained, the condition of the soil changed.
Mumbai spirit			17 people died today in Chembur…rainy season has just started..people are crying their houses are filled with dirty water..cars are floating…mumbai is flooding…<br><br>Mumbai is running only on Mumbaikars spirit!!
Event		This code refers to Tweets that provided an informational update about an event that will take place/took place.	Panel Discussion on Chitale Fact Finding Report 2006 on Mumbai Rivers and Infrastructure<br>The event is remembering the catastrophic incident of the July 26, 2005 Mumbai floods<br>When: Sat, July 31 2021 at 4:30PM<br>Facebook Live- https://t.co/kjLkBZmdvp<br>#river #infrastructure #26July<br>https://t.co/Mi5leQaXI7
Call to action	Request support	This sub-code is assigned to Tweets that express a request for support in view of the impacts of flooding.	On July 16, 2020, i was near the building after collapsed due to heavy rain and also the building was in detoriated condition that led to this tragedy of 20+ dead. Now the rainy season is due in July requesting @mybmc, @mayor_mumbai to please take care of detoriarating buildings. https://t.co/EwXpU9lCwj
	Call to action	This sub-code refers to Tweets which express a call for action on flood risk	Maharashtra floods: Mumbai has some of richest people in world, they should help, says Sanjay Raut | Mumbai News - Times of India https://t.co/R8kMjbc0GE

		management (including preparedness and relief)	
Covid		This code is assigned to Tweets that refer to management and/or impact of the ongoing Covid pandemic on flood risk preparedness and management.	CM Uddhav Balasaheb Thackeray has directed the state administration to be alert as a 4-day heavy rainfall prediction has been issued for Mumbai & Konkan. The treatment of patients must not be affected &, if needed, shift citizens from vulnerable establishments to safer places.
Complaints	Radar	This sub-code refers to Tweets while complain about the functioning and use of the Doppler radars in monsoon weather predictions.	Mumbai is vulnerable to extreme rain events during monsoon so for that purpose local Doppler radar is very important to warn people. It's been over 3 weeks IMD Mumbai Radar not working. Local media needs to cover this story. This has happened way too many times last few years.
	Ecosystems	This sub-code refers to Tweets which express complaints about the condition of ecosystem protection.	16 years on, Brimstowad drainage system still unfinished.<br><br>Wetlands of Mumbai are concretized, mangroves are being uprooted. Walls are built in our rivers and natural nullahs.<br><br>Coastal road is just one of the reasons. The more reclamation, more water will keep clogging Mumbai. https://t.co/5acyKJ32XN
	Potholes	This sub-code refers to Tweets which complain about potholes on roads.	Hard to believe but true. ₹48 crore spent on filling 33,000 potholes this monsoon in Mumbai. ₹14,000 spent on fixing each pothole. But roads full of potholes even now. https://t.co/BmhQLOmxEd @VinodMishra4U @mihirkotecha @rais_shk @AmeetSatam @ShelarAshish
	Electricity shortage	This sub-code refers to Tweets which complain about electricity shortage as an impact heavy rainfall.	In last 2.5hrs it has rained 127mm between 9.30pm to 12am. All from b2b #Thunderstorm formation⛈<br><br>No #electricity in #Badlapur #west from 11pm.. #Winds gust also touched 68km/hr.
	Building collapse	This sub-code refers to Tweets which complain about the conditions of buildings leading to building collapses during heavy rainfall in the monsoon.	On July 16, 2020, i was near the building after collapsed due to heavy rain and also the building was in detoriated condition that led to this tragedy of 20+ dead. Now the rainy season is due in July requesting @mybmc, @mayor_mumbai to please take care of detoriarating buildings. https://t.co/EwXpU9lC
	Drainage	This sub-code refers to Tweets which complain about the condition and cleaning of the drainage system before the monsoon.	Tax payers money down the drain? In 10 years, BMC spent Rs 7000 crore on desilting, Storm Water Drain works reveals RTI filed by BJP MLA AmeetSatam. ₹1000 crore spent on nullahsafai alone but Mumbai still floods @AUThackeray @mihirkotecha https://t.co/MR26fYbxYo @chaitanya_pm
	Waterlogging	This sub-code refers to Tweets which complain about the impacts of	Vasai: 70-year-old man sits in flooded water to protest against waterlogging in his ground-floor flat https://t.co/NJ4GI5PNgx

		waterlogging as a result of flooding.	
	Exams	This sub-code refers to Tweets that complain about the conditions for being allowed to write important exams in the event of heavy rainfall and flooding. In some Tweets, actors describe impacts of flooding and related difficulties which pose challenges for students to prepare and appear for exams.	They reached 20 min late , due to heavy rainfall. Exam was scheduled at 10 AM.<br><br>@CMOMaharashtra @PawarSpeaks @AjitPawarSpeaks<br>Please allow them to appear in the ESE exam. Their life and 2 years preparation is on stack...<br>Maharshi Dayanand College Parel Mumbai.<br>#ESE2021 https://t.co/cLWIdaS3eD
	General preparedness	This sub-code refers to Tweets which complain about the overall state of preparedness towards the annual monsoons and heavy rain-related impacts including flooding.	First spell of #mumbairain's and see the preparedness of @mybmc<br>Visuals of Dadar . https://t.co/IhIM0viuIQ
	Manholes	This sub-code refers to Tweets which complain about the open manholes that pose severe threats during floods.	Two women walking along a flooded footpath in #Mumbai fell into an open manhole evoking outrage from city residents.<br><br>https://t.co/96E97gGu3J
	Media	This sub-code refers to Tweets which complain about the role of media and coverage of flooding related issues.	I strongly believe that natural disasters which happen (vastly more frequently) in places that aren't the US and Europe should receive equal coverage<br><br>The question is why so many people in the US and Europe don't care about the rest of the world https://t.co/lsZhEVGhmp
	Reclamation	This sub-code refers to Tweets which complain about the impacts of reclamation on flood risk.	Mumbai flooded?<br>Mithi overflowing?<br><br>If we tinker with floodplains what else can we expect?<br><br>This is Aarey Metro Depot plot where natural floodplain was filled up<br>A plot that was lower in level than Mithi river (zoom in)<br><br>The result of filing up floodplains is for all to see today https://t.co/Vr7qjwPyRX
Locations mentioned		In the absence of geo-referenced Tweets, this code was assigned to Tweets which contain the name of a location.	Andheri Subway waterlogged in #Mumbai as rain continues to lash the city.<br><br>(Video credit: ANI)<br><br>#MumbaiRains<br>#MaharashtraRains https://t.co/1ANzjsohpN

Transport	Not affected	This sub-code was included in the first round of coding for transport infrastructure that was not affected due to flooding.	
	Boat	This sub-code refers to transport-related Tweets which provide an update on water/boat transport	In a few weeks, Navi Mumbai may just be a #watertaxi ride away from south Mumbai.<br><br>The Maharashtra Maritime Board (MMB) will be holding a trial run of water taxi service between Belapur in #NaviMumbai & Mumbai in October.<br><br>@IndianExpress<br><br>@maha_tourism<br><br>https://t.co/zBTJSbofw4
	Railways	This sub-code refers to Tweets which share updates, impacts and preparations for the functioning of Mumbai's railway services – the lifeline of the city.	Reviewed monsoon preparedness of Mumbai Suburban for making a roadmap & precautionary plan.<br><br>Examined current status of vulnerable areas & devised a plan for smooth functioning of trains.<br><br>We are committed to ensure no inconvenience is caused to Mumbaikars as monsoon begins. https://t.co/TVW53U7noR
	Bus	This sub-code refers to Tweets which provide updates on the impact of flooding on bus travel	Mumbai: Due to heavy rain and waterlogging in low-lying areas, buses have been diverted. https://t.co/If8JMzkRzh
	Solutions	This sub-code refers to Tweets which share transport-related measures in adaptation to flooding	To make the crucial road transport route floodproof, the Maharashtra government plans to ask the National Highway Authority of India to build flyovers on the Mumbai-Bengaluru highway.<br><br>https://t.co/xtTnOTr1Qd
	Traffic update	This sub-code refers to Tweets which provide traffic-related updates due to the impact of flooding.	#Mumbai Traffic Police says #Andheri Subway will remain closed every day from 10 pm to 6 am (21st June- 30th September), for traffic to avoid any accident or loss of life due to waterlogging<br><br>#MumbaiRains
	Flights	This sub-code refers to Tweets which provide information on the impact of flooding on flight travel	#6ETravelAdvisory: Due to waterlogging in some parts of #Delhi and #Mumbai, we advise passengers to keep enough travel time in hand while travelling to the airport. To check your flight status please visit https://t.co/TQCzzy2a2s. Stay Safe!
	Access	This sub-code refers to Tweets which show the impact of flooding on access to services dependent on transport infrastructure.	Gokul largest milk brand will not be able to supply milk to Mumbai on Saturday. Flooding in parts of the state --Kolhapur, Sangli, Konkan & closure of state & N.highways has affected the transportation & Milk collection #MaharashtraRains<br>#MumbaiRains<br>@fpjindia
	Roads	This sub-code refers to Tweets which	Hello Coastal road fans.

		highlight the impact of road infrastructure on flooding as well as the impact of flooding on roads.	This is the situation in Mumbai with just few hours of rainfall today.<br>We wonder what would be the fate of the road so close to sea & that underneath via tunnels.<br>Will it be viable for cars or boats/ submarines□<br>#SaveOurCoast<br>#CoastalRoad #MumbaiRains<br>https://t.co/cXnQ4BBfkp
Response		This code refers to Tweets which describe a response measure taken or suggested in view of flood risk mitigation.	The pumping station at Mogra nullah, with a capacity of pumping out 42,000 litre water per second, is all set to be made, and the pumping station at Mahul nullah is awaiting few clearances from GoI https://t.co/pe17wcOGAp via @IndianExpress
Preparedness		This code refers to Tweets which describe a response measure taken or suggested in view of flood risk preparedness.	Considering IMD's heavy rainfall forecast for the next 4-5 days in Mumbai, CM Uddhav Balasaheb Thackeray has directed @mybmc & all agencies to be prepared for any eventualities, work around the clock in close co-ordination to tackle them. All rescue teams must be on stand-by.
Luxury problem		This code is assigned to Tweets which describe luxury problems related to impact of flooding.	Daughter - if it was offline school would have surely got a holiday today. Hope atleast some teachers face network issues to compensate for the lost holiday. 😂😂😂<br><br>#mumbairain
Impact		This code refers to Tweets which describe impacts of flooding, mostly in terms of deaths.	"More than 30 people have died in the Indian city of Mumbai after an intense burst of rainfall caused a landslide and wall collapse, as changing monsoon patterns due to climate change lead to more extreme rains across India."<br><br>https://t.co/ekyVzwV0pc
Sympathy		This code refers to Tweets expressing sentiments of sympathy towards impacts of floods on humans and animals.	Pained to know about house & wall collapse incidents in #Chembur , Vikhroli & Bhandup in Mumbai.<br>Heartfelt condolences to families who lost their loved ones.<br>Praying for the speedy recovery of the injured. #MumbaiRains
Water supply		This code refers to Tweets which state the impact of flooding and heavy rainfall on water supply in the city.	Due to the inundation of rain water in the Bhandup Water Purification Complex, the Filtration and Pumping plants at the complex had to be shut down as a precautionary measure. Due to this, water supply in most of the parts of Mumbai has been disrupted today (July 18, 2021)
Corruption		This code refers to Tweets which suggest the corruption involved in measures related to flood risk management	Rain, rain, go away...<br>Global Tenders are on the way...<br>Come again another day...<br>Meanwhile PR is on the sway !!!<br><br>#MumbaiRains<br>#Corrupt_Mcgm https://t.co/MJJsCF5rbl
Philanthropic action		This code refers to Tweets which describe philanthropic measures taken towards individuals affected by floods.	Bengali news paper article<br>- 70 new houses by Salman Khan for flood affected victims in #Mumbai<br><br>Man with golden heart @BeingSalmanKhan<br>https://t.co/jdo1K8vPAy
